# A Naturalistic Study of the Acceptability and Effectiveness of Internet-Delivered Cognitive Behavioural Therapy for Psychiatric Disorders in Older Australians

**DOI:** 10.1371/journal.pone.0071825

**Published:** 2013-08-12

**Authors:** Louise Mewton, Perminder S. Sachdev, Gavin Andrews

**Affiliations:** 1 Centre for Healthy Brain and Ageing (CHeBA), School of Psychiatry, University of New South Wales, Sydney, Australia; 2 Clinical Research Unit for Anxiety and Depression (CRUfAD), St Vincent’s Hospital, Sydney, Australia; 3 Neuropsychiatric Institute, Prince of Wales Hospital, Sydney, Australia; University of Iowa Hospitals & Clinics, United States of America

## Abstract

**Objectives:**

The current study investigates the acceptability, effectiveness and uptake of internet-delivered cognitive behavioural therapy (iCBT) amongst older individuals (>60 years) seeking psychiatric treatment in general practice.

**Methods:**

The sample consisted of 2413 (mean age 39.5; range 18–83 years) patients prescribed iCBT through This Way Up clinic by their primary care clinician. The intervention consisted of six fully automated, unassisted online lessons specific to four disorders major depression, generalised anxiety disorder, panic disorder or social phobia. Patients were categorised into five age groups (18–29 years, 30–39 years, 40–49 years, 50–59 years, 60 years and above). 225 (9.3%) patients were aged over 60 years. Analyses were conducted across the four disorders to ensure sufficient sample sizes in the 60 years and older age group. Age differences in adherence to the six lesson courses were assessed to demonstrate acceptability. Age-based reductions in psychological distress (Kessler Psychological Distress Scale; K10) and disability (the World Health Organisation Disability Assessment Schedule; WHODAS-II) were compared to demonstrate effectiveness. To evaluate the uptake of iCBT, the age distribution of those commencing iCBT was compared with the prevalence of these disorders in the 2007 Australian National Survey of Mental Health and Well-Being.

**Results:**

Older adults were more likely to complete all six lessons when compared with their younger counterparts. Marginal model analyses indicated that there were significant reductions in the K10 and WHODAS-II from baseline to post-intervention, regardless of age (*p*<0.001). The measurement occasion by age interactions were not significant, indicating that individuals showed similar reductions in the K10 and WHODAS-II regardless of age. In general, the age distribution of individuals commencing the iCBT courses matched the age distribution of the four diagnoses in the Australian general population, indicating that iCBT successfully captures older individuals who need treatment.

**Conclusion:**

iCBT is effective and acceptable for use in older populations.

## Introduction

There is a world-wide trend for an ageing of our populations. The proportion of Australians aged 65 years or over is predicted to increase from 13% (1.1 million) in 2010 to 23% (8.1 million) in 2050 [1]. Epidemiological surveys of the Australian population suggest that approximately 1 in 6 individuals over the age of 65 is diagnosed with a mental disorder in the 12 months prior to interview [2], with similar rates established internationally [3]. These statistics suggest that geriatric mental illness is common, and, in the future, the number of older adults living with mental illness will increase dramatically. One of the current challenges for global public health is the identification of mental health interventions that are effective and acceptable to older populations, and that have the potential for widespread dissemination.

Over recent years there has been a proliferation of internet delivered medical interventions across a wide range of clinical conditions. Randomised controlled trials have demonstrated the efficacy of internet delivered treatments, particularly those based on cognitive behavioural therapy (iCBT). Reviews of the literature indicate that iCBT is highly efficacious across a range of mental and physical disorders, with benefits in terms of patient accessibility and convenience [4]–[6]. Given the efficacy, cost effectiveness, scalability and adaptability of iCBT, the potential for widespread dissemination of these treatments is vast. Recent research has focused on the effectiveness of iCBT in practice [7]–[10]. These studies have shown that when iCBT is disseminated into practice, reductions in psychiatric symptoms and psychological distress are considerable, and commensurate with those achieved in randomised controlled trials. However, the acceptability, effectiveness and uptake of iCBT treatment for mental disorders has not been established in older populations [5], [6]. Given that rates of internet use vary considerably by age [11], age-based differences in the adoption of internet delivered treatments should be investigated.

In view of this, the current study aims to:

1. Investigate relative iCBT adherence rates amongst younger and older adults;
2. Establish whether younger and older adults gain similar treatment benefits from iCBT; and
3. Determine whether age-based patterns of enrolment for iCBT reflect the age distribution of mental illness in the general population.

The current study addresses these questions in data collected as part of routine quality assurance activities. The interventions to be assessed have been shown to be efficacious in several randomised controlled trials [12]–[17]. On the basis of these findings, these interventions were disseminated into general practice across Australia in 2010. This naturalistic study is based on the 2413 patients who were prescribed one of these interventions by their primary care physician since dissemination in October 2010.

## Methods

### Participants

Between October 2010 and January 2013, 2413 (mean age 39.5; range 18–83 years) patients had been prescribed iCBT for major depression, generalised anxiety disorder (GAD), panic disorder or social phobia through This Way Up clinic (www.thiswayup.org) by their primary care clinician. Of these patients, 225 (9.3%) were aged over 60 years. Information was aggregated across all four disorders (major depression, GAD, panic disorder and social phobia) to ensure sufficient cell sizes to conduct analyses in the older age group. Prescribing clinicians were advised that patients were unlikely to benefit if they had persistent suicidal thoughts, drug or alcohol dependence, schizophrenia, bipolar disorder, or were on atypical antipsychotics or benzodiazepines. Data were confined to routine measures (described below) used to inform practitioners about the progress of their patients. This study was approved as part of the quality assurance activities undertaken by the Patient Safety and Quality Unit at St. Vincent’s Hospital, Sydney, with whom a copy of this manuscript has been lodged. Prior to enrolment in any of the treatment programs, all individuals are informed that data will be collected and used as per the following: ‘*By participating in This Way Up clinic, you acknowledge that your data will be pooled, analysed and periodically published in scientific articles to enhance scientific knowledge in anxiety and depression. In any publication, information will be provided in such a way that you cannot be identified’*. All patients provided electronic informed consent that their pooled data could be used for these purposes.

### Intervention/Procedure

Each iCBT course consisted of six fully automated, unassisted online lessons involving components such as psycho-education, behavioural activation, cognitive restructuring, problem solving, graded exposure, relapse prevention, and assertiveness skills. Content was presented in the form of an illustrated story in which the character gains mastery over their symptoms with the help of a clinician. The patient followed the character’s journey to recovery across the 6 lessons. At the end of each lesson the patient downloaded “homework” tasks which reinforced the content of the lesson.

### Measures

At baseline, limited demographic information (age, gender) was collected for each patient and rurality was imputed from the location of the prescribing clinician. The current study focuses on two measures that were administered across all four courses: the Kessler Psychological Distress Scale (K-10) and the World Health Organisation Disability Assessment Schedule (WHODAS-II).

The K10 is a ten item dimensional scale developed as a measure of non-specific psychological distress [18]. Scores are aggregated across the responses, and range from 10–50 with higher scores suggesting higher levels of non-specific psychological distress and severity of psychiatric illness. The K-10 has good psychometric properties [18], demonstrating high sensitivity and specificity for current anxiety and affective disorders [19]. Patients completed the K-10 before each of the six lessons.

The WHODAS-II is a health-related quality of life instrument that assesses functioning and disability [20]. Scores range from 12 to 60, with higher scores suggesting a greater level of disability. The WHODAS-II has demonstrated good validity as a measure of disability and functioning [21]. The WHODAS-II was completed at baseline and prior to the final lesson.

Patients also completed disorder-specific assessment measures at baseline and prior to the final lesson. However, the small number of older individuals within each specific course precluded analyses of these disorder-specific outcomes.

### Statistical Analysis

To investigate relative adherence amongst younger and older adults, adherence (i.e., completion of all 6 lessons) was cross-tabulated with the five category age variable (8–29 years, 30–39 years, 40–49 years, 50–59 years and 60+ years). Chi-square tests were conducted in SPSS version 19 to determine whether adherence and baseline characteristics differed significantly by age, whilst two-proportion z-scores were used to determine which age groups differed significantly from those aged over 60 years.

Previous research of This Way Up clinic data has indicated that approximately 60% of patients complete all 6 courses [7]–[10], a much higher level than the levels of adherence in open access research settings, which are typically very low, ranging from around 1% [22] to 10% [23]. Notwithstanding this, we used analytic methods that made use of all the available data without biasing parameter estimates. Marginal models estimate the regression coefficients in repeated measures studies with unbalanced data using maximum likelihood estimation, making use of the incomplete data in a way that does not bias the parameter estimates [24]. To investigate effects on psychological distress (K10) and functioning (WHODAS-II) from pre- to post-treatment, a marginal linear model was implemented using the MIXED procedure with a repeated statement in SPSS Version 19. Measurement occasion (pre-post) and age (categorised as 18–29 years, 30–39 years, 40–49 years, 50–59 years and 60+ years) were treated as categorical variables and an unstructured (UN) covariance structure was specified to model the relationship amongst observations at different time points. Interactions between measurement occasion and the categorical age variable were calculated to indicate whether treatment effects over time differed as a function of age. Estimated marginal means, which indicate the mean response for each categorical variable adjusted for any other variables in the model (i.e., time, age and the time by age interaction) were also calculated.

To investigate the penetration or uptake of iCBT amongst older individuals, the age distribution of individuals enrolling in the individual courses was compared with the age distribution of individuals diagnosed with 12-month major depression, GAD, panic disorder and social phobia in the Australian population. General population prevalence data was obtained from the 2007 Australian National Survey of Mental Health and Well-Being, an epidemiological survey of 8841 community-dwelling adults. This data was weighted according to census benchmarks to match the age and gender distributions of the Australian population. More information of the 2007 National Survey is provided elsewhere [25]. Frequencies of disorders in the general population were calculated using weighted data in SAS 9.2 in order to adjust for the complex survey design. Two-proportion z-scores were then calculated to determine whether the proportion of older individuals prescribed each of the iCBT courses differed significantly to the proportion of older individuals diagnosed with each of the target disorders in the Australian population.

## Results

Table 1 lists the baseline characteristics of individuals prescribed the iCBT courses by age. When compared with those aged over 60 years, those in the 50–59 year age group were more likely to be based in a rural location, whilst those in the 30–39 year age group were more likely to live in an urban residence. Those aged over 60 years were more likely to complete all 6 lessons when compared with each of the younger age groups, except for those in the 50–59 year age group. Age groups were similar in terms of sex and prescribing clinician.

**Table 1:** Baseline characteristics and adherence of individuals commencing iCBT courses for major depression, generalised anxiety disorder, panic disorder and social phobia by age.

	Whole sample	18–29 years	30–39 years	40–49 years	50–59 years	60+ years	Overall Chi-Square
	(n = 2413)	(n = 681)	(n = 600)	(n = 512)	(n = 394)	(n = 225)	
	n (%)	n (%)	n (%)	n (%)	n (%)	n (%)	
Female	1554 (64.4)	471 (69.2)	377 (62.8)	327 (63.9)	245 (62.2)	134 (59.6)	χ^2^(4, *N* = 2413) = 10.60, *p* = .03
Rural	1046 (43.3)	282 (41.3)	227 (37.8)^a^	237 (46.3)	209 (53.0)^a^	91 (40.4)	χ^2^(4, *N* = 2413) = 26.20, *p*<.001
Clinician							
GP	1207 (50.0)	336 (49.3)	306 (51.0)	306 (51.5)	263 (47.0)	117 (52.0)	χ^2^(8, *N* = 2413) = 7.38, *p* = .50
Psychologist	712 (29.5)	191 (28.0)	173 (28.8)	148 (29.0)	134 (34.0)	66 (29.3)	
Other	493 (20.4)	155 (22.7)	121 (20.2)	100 (19.6)	75 (19.0)	42 (18.7)	
Completed course	1261 (52.3)	267 (39.1)^a^	292 (48.7)^a^	286 (55.9)^a^	258 (65.5)	158 (70.2)	χ^2^(4, *N* = 2413) = 109.46, *p*<.001

a Proportion significantly different when compared with the 60+ years age group (after Bonferroni correction for multiple comparisons).

Table 2 displays the results from the marginal models with K10 and WHODAS-II scores as separate continuous outcome variables. In each model, the main effect of time was statistically significant (*p*<0.001 in both cases), indicating that psychological distress decreased and functioning increased significantly following iCBT. The interactions between time and age were not statistically significant, indicating that scores on both the K10 and WHODAS-II reduced at a similar rate for both age groups.

**Table 2:** Estimated marginal K10 and WHODAS-II means and results from the marginal model with K10 and WHODAS-II scores as the outcome variables.

	Estimated Marginal Mean K10 Score		Marginal Model			
	Baseline (SE)	Post-treatment (SE)	Beta (SE)	df	t	*p*
Time	27.6 (0.2)	19.5 (0.2)	7.6 (0.5)	1368	14.8	<0.001
Age*Time						
18–29	28.9 (0.3)	20.5 (0.4)	0.8 (0.6)	1388	1.3	0.21
30–39	27.2 (0.3)	19.1 (0.4)	0.5 (0.6)	1382	0.7	0.47
40–49	27.5 (0.3)	18.8 (0.4)	1.1 (0.6)	1378	1.7	0.09
50–59	27.7 (0.4)	19.9 (0.4)	0.2 (0.7)	1372	0.3	0.75
60+	26.8 (0.5)	19.1 (0.6)	–	–	–	–
	**Estimated Marginal Mean WHODAS-II Score**		**Interactions with Time**			
	**Baseline (SE)**	**Post-treatment (SE)**	**Beta (SE)**	**df**	**t**	***p***
Time	27.0 (0.2)	22.8 (0.2)	4.3 (0.5)	1301	8.5	<0.001
Age*Time						0.82
18–29	27.2 (0.3)	23.1 (0.4)	−0.1	1315	−0.2	0.37
30–39	25.5 (0.3)	21.8 (0.4)	−0.6	1312	−0.9	0.45
40–49	27.1 (0.4)	22.3 (0.4)	0.5	1308	0.8	0.71
50–59	28.0 (0.4)	23.9 (0.5)	−0.2	1304	−0.4	0.82
60+	27.3 (0.6)	23.0 (0.6)	–	–	–	–

Table 3 compares the age distribution of individuals initiating iCBT through This Way Up clinic with the age distribution of individuals meeting 12-month criteria for major depression, GAD, panic disorder and social phobia in the Australian population. When compared to those meeting criteria for social phobia in the Australian population, there were significantly more 18–29 year olds enrolling in the social phobia iCBT course. Otherwise, the age distribution of those enrolling in the iCBT courses matched the age distribution of those meeting criteria for each of the disorders in the Australian population.

**Table 3:** Age distribution of individuals enrolling in the This Way Up Clinic iCBT courses and of those diagnosed with psychiatric disorders in the general population according to the 2007 Australian National Survey of Mental Health and Well-Being (NSMHWB).

Disorder	Age group (years)	This Way Up Clinic (n = 2413 N (%)	NSMHWB (n = 8841)N (%)	Two-ProportionZ-score (*p*)
Major Depression	18–29	244 (24.2)	167 (25.5)	0.30 (0.77)
	30–39	227 (22.5)	152 (23.7)	0.27 (0.79)
	40–49	244 (24.2)	149 (26.8)	0.57 (0.57)
	50–59	183 (18.2)	117 (14.3)	0.90 (0.37)
	60+	110 (10.9)	78 (9.7)	0.27 (0.79)
Generalised Anxiety Disorder	18–29	233 (27.3)	109 (18.5)	1.85 (0.06)
	30–39	231 (27.0)	108 (21.7)	1.07 (0.28)
	40–49	176 (20.6)	115 (29.4)	1.68 (0.09)
	50–59	141 (16.5)	103 (18.9)	0.48 (0.63)
	60+	73 (8.6)	70 (11.6)	0.59 (0.55)
Panic Disorder	18–29	76 (27.1)	224 (30.0)	0.48 (0.63)
	30–39	82 (29.3)	155 (22.9)	1.05 (0.29)
	40–49	56 (20.0)	122 (22.9)	0.44 (0.66)
	50–59	37 (9.4)	94 (14.4)	0.82 (0.41)
	60+	29 (10.4)	85 (9.8)	0.09 (0.93)
Social Phobia	18–29	129 (47.6)	182 (28.9)	3.37 (<0.001)
	30–39	60 (22.1)	146 (23.3)	0.19 (0.85)
	40–49	36 (13.3)	117 (24.0)	1.53 (0.13)
	50–59	33 (8.4)	103 (16.9)	1.38 (0.17)
	60+	13 (4.8)	64 (6.8)	0.29 (0.77)

## Discussion

These findings indicate that iCBT prescribed across a range of psychiatric disorders is effective and acceptable amongst older treatment-seeking populations. Reductions in psychological distress and improvements in functioning were significant and similar across age groups. Adherence, on the other hand, was more likely among older individuals when compared with their younger counterparts. The age distribution of those commencing iCBT treatment also matched the age distributions of those diagnosed with these disorders in the general population, indicating that iCBT successfully captures older individuals who need treatment.

This study was conducted within a naturalistic framework, which means that the external validity of these findings was maximised. However, these findings need to be interpreted within the context of some limitations. It was not possible to establish whether treatment effects were sustained over time due to the lack of follow up data. The lack of formal exclusion criteria also meant that patients may have been using adjunctive treatments which contributed to the magnitude of treatment effects.

When considering the older population, there are some barriers to the successful treatment of illness over the internet. For instance, older age groups report lower rates of internet use when compared with their younger counterparts [26]. Age-related issues, such as impairments in vision, manual dexterity and cognition have been identified as factors that may prevent the uptake of technology-based health interventions [27]. This has led to the popular belief that online treatments may not be suitable for older individuals, or that older individuals may be less willing to commence technology-based treatment. Reviews of iCBT for mental illness have indicated that older individuals are generally under-represented in the samples that provide efficacy data for these programs [5], [6]. This has precluded subgroup analyses based on age, however, positive and large effects for these interventions are often elicited after adjusting for age, providing preliminary evidence that these programs are effective across the age spectrum. Consistent with the current results, previous research has also indicated that older, compared with younger, individuals are more likely to adhere to these programs when completed under the care of their primary care physician [7], [10]. Taken together, these previous findings suggest that while iCBT interventions may be effective and acceptable regardless of age, the reach of these programs amongst older populations may be limited. The current study provides further evidence that online treatments for psychiatric illness are both effective and acceptable for older treatment-seeking patients. Importantly, the current findings also indicate that, when disseminated into general practice, the proportion of older adults commencing these programs is consistent with what would be expected.
